# Strong Costs and Benefits of Winter Acclimatization in *Drosophila melanogaster*

**DOI:** 10.1371/journal.pone.0130307

**Published:** 2015-06-15

**Authors:** Mads Fristrup Schou, Volker Loeschcke, Torsten Nygaard Kristensen

**Affiliations:** 1 Department of Bioscience, Aarhus University, Aarhus C, Denmark; 2 Department of Chemistry and Bioscience, Aalborg University, Aalborg, Denmark; Clemson University, UNITED STATES

## Abstract

Studies on thermal acclimation in insects are often performed on animals acclimated in the laboratory under conditions that are not ecologically relevant. Costs and benefits of acclimation responses under such conditions may not reflect costs and benefits in natural populations subjected to daily and seasonal temperature fluctuations. Here we estimated costs and benefits in thermal tolerance limits in relation to winter acclimatization of *Drosophila melanogaster*. We sampled flies from a natural habitat during winter in Denmark (field flies) and compared heat and cold tolerance of these to that of flies collected from the same natural population, but acclimated to 25 °C or 13 °C in the laboratory (laboratory flies). We further obtained thermal performance curves for egg-to-adult viability of field and laboratory (25 °C) flies, to estimate possible cross-generational effects of acclimation. We found much higher cold tolerance and a lowered heat tolerance in field flies compared to laboratory flies reared at 25 °C. Flies reared in the laboratory at 13 °C exhibited the same thermal cost-benefit relations as the winter acclimatized flies. We also found a cost of winter acclimatization in terms of decreased egg-to-adult viability at high temperatures of eggs laid by winter acclimatized flies. Based on our findings we suggest that winter acclimatization in nature can induce strong benefits in terms of increased cold tolerance. These benefits can be reproduced in the laboratory under ecologically relevant rearing and testing conditions, and should be incorporated in species distribution modelling. Winter acclimatization also leads to decreased heat tolerance. This may create a mismatch between acclimation responses and the thermal environment, e.g. if temperatures suddenly increase during spring, under current and expected more variable future climatic conditions.

## Introduction

Climate change increases global mean temperatures and temperature variability, leaving thermal environments more extreme [1–3]. One aim in the fields of evolutionary physiology and ecology is to understand how and if animals and plants are able to respond to these changes by adaptive phenotypic plasticity. Plastic changes in morphological or physiological traits induced by acclimation (response to controlled experimental changes) or acclimatization (response to natural climatic conditions) can significantly increase cold and heat tolerance [4,5], and under some circumstances enable individuals to cope with thermal conditions that would otherwise be lethal [6–8]. Thus phenotypic plasticity is an important response to daily and seasonal temperature fluctuations and may be an effective short-term response to future climate change. Recent studies suggest that the current and future distribution of ectotherms is highly dependent on their upper and lower thermal limits [9]. Therefore studies investigating the plasticity of these limits are needed, if we are to use such data to increase our understanding of how natural populations thrive in their natural habitat and how climate change may affect species distributions.

The physiological changes induced by exposure to suboptimal temperatures have been widely studied in insects [4,5,10–14]. In temperate climatic zones physiological acclimatization in ectotherms to cold conditions is important for overwintering abilities [15–17]. However studies on cold acclimation in ectotherms are typically performed in the laboratory on populations adapted to the laboratory [4,5,12,18], and can be criticized for lack of ecological relevance, and may therefore not be generalised to field conditions [19–21]. Effects of cold acclimation in insects, tested in the laboratory, typically provide evidence for strong benefits in the form of increased cold tolerance, but contrary to what might be expected, costs in terms of decreased heat tolerance in cold acclimated insects are usually not observed [4,5,12,18]. However, when assessed under field conditions, cold acclimatization can lead to strong benefits but also strong costs, if temperatures are high and *vice versa* with heat acclimatization [7,22–24]. This illustrates that laboratory investigations may not always correctly portray the ecological parameters important for acclimatization responses of insects in the wild. The advantages of performing carefully controlled laboratory experiments instead of field experiments are many (e.g. good control of environmental parameters), but to fully appreciate these advantages and eventually increase our understanding of the importance of thermal acclimation/acclimatization for fitness in nature, improvements in the ecological relevance of laboratory experiments are necessary.

In previous field studies on the effects of thermal acclimation, the acclimation treatment has typically been performed in the laboratory, and effects have been tested in the field or under semi-natural settings [7,22,23]. Here ‘we bring the field to the laboratory’, and aim to estimate to what extent *D*. *melanogaster* acclimatize to colder temperatures in the winter at the natural habitat in temperate Denmark, and whether this acclimatization can be replicated in the laboratory. Thermal tolerance assessments of temporal collections from a natural population from fall to spring would provide valuable information on the change in thermal tolerance across seasons, but recent studies suggest strong seasonal selection [25,26], whereby temporal changes in thermal tolerances may be attributed to genetic adaptation and not only acclimatization. Another challenge in using temporal samples across seasons is the inability to assess cold and heat tolerance of different groups of flies simultaneously, which challenges the interpretation of data. For these reasons we choose to focus on the beginning of winter, at which flies can be assumed to have been acclimatized to the coming cold climate throughout the fall. We assessed if winter acclimatization leads to an increased cold tolerance (2 h chill coma recovery, critical thermal minimum), and whether this acclimatization has a cost in terms of decreased heat tolerance (heat knock down, critical thermal maximum). Cross-generational effects can be an important component of thermal acclimatization and are often overlooked in studies of thermal adaptation [27]. Hence, we further obtained thermal performance curves for egg-to-adult viability, based on eggs laid by flies reared at 25°C in the laboratory and field flies, to estimate possible cross-generational effects of winter acclimatization. In early December we collected flies from an orchard apple compost heap. We immediately performed laboratory thermal assays and compared the performance to flies collected from the same natural population, but acclimated to the laboratory at 13 or 25°C. The rearing temperature of 25°C served as a benign control, while 13°C mimicked the average temperature in the microhabitat of the natural population in December. If the observed thermal cost-benefit relations between field and laboratory flies reared at 25°C are mainly a consequence of the differing thermal regimes, and thus different thermal acclimation/acclimatization, we expect the laboratory flies reared at 13°C to perform similar to the field flies.

## Results

### Habitat

**Fig 1:**
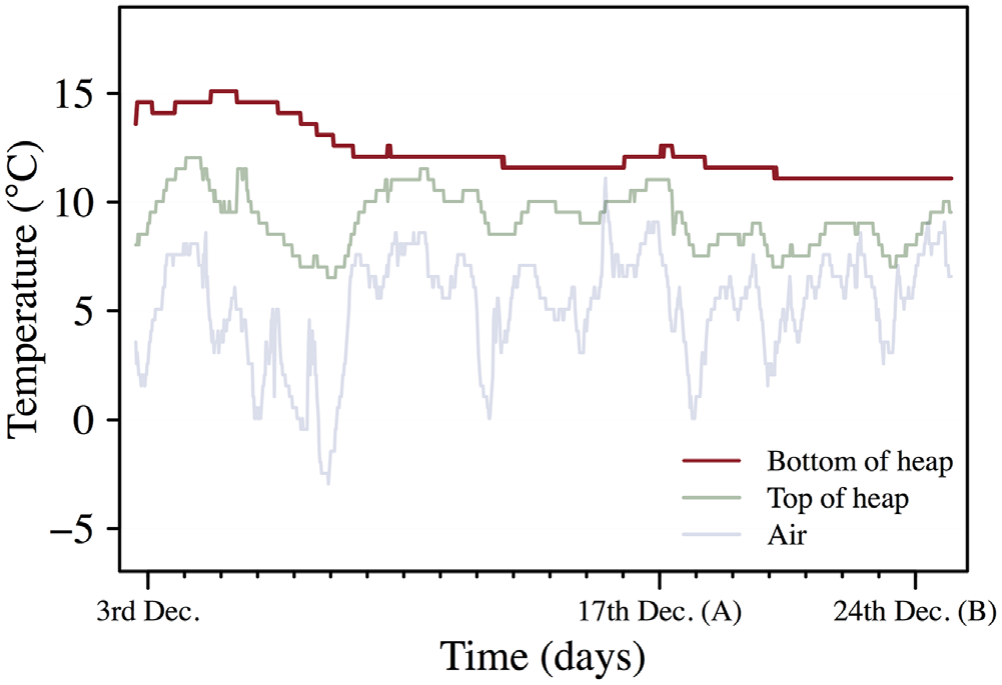
Field site temperatures. Air temperature 1 m above the heap (air), temperature 10 cm below the surface of the heap (top of heap) and temperature 40 cm below the surface of the heap (bottom of heap) at the field collection site. The temperatures were logged every half hour. (A) Cold and heat tolerance were assessed; (B) Egg-to-adult viability experiment was performed.

The natural population used in this study was obtained from a 2 m (length) * 2 m (width) * 0.5 m (depth) apple compost heap which comprises a typical habitat for *D*. *melanogaster* in temperate zones. The fermentation process in the compost increases the temperature during fall and winter to approximately 8°C above air temperature, and buffers the daily variation in air temperature (Fig 1). Under laboratory conditions *D*. *melanogaster* can complete the development from egg to adult at mean temperatures down to approximately 10°C [28,29], which is similar to the early winter temperatures recorded in the heap (Fig 1).

### Thermal Tolerance Assays

**Fig 2:**
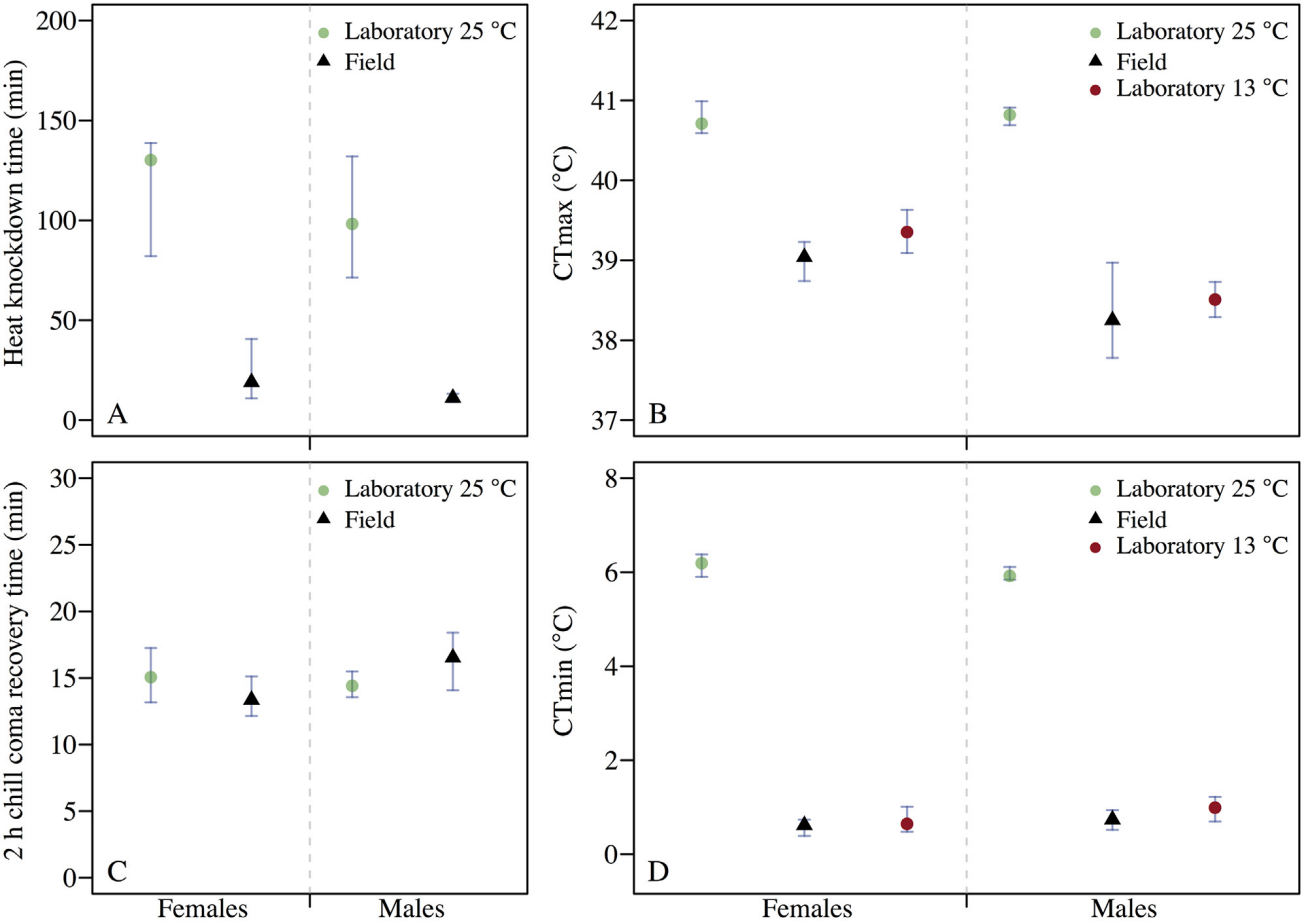
Thermal tolerance assays. Medians with 95% confidence intervals (obtained with bootstrapping; *n* = 10,000) for stress tolerance of male and female flies from the laboratory reared at 25°C and the field in four temperature stress assays: (A) heat knockdown; (B) heat ramping; (C) 2 h chill coma recovery; (D) cold ramping, *n* = 22–25. Heat and cold ramping were also performed on laboratory flies reared at 13°C.

Freshly collected flies from the compost heap (field flies) and flies reared in the laboratory for two generations at 25°C were tested for heat tolerance, using the assays ‘static heat knockdown’ and ‘heat ramping’, and for cold tolerance, using the assays ‘2 h chill coma recovery’ and ‘cold ramping’. We found a significantly lower heat knockdown time for males and females from the field, compared to the laboratory flies reared at 25°C (Table 1; Fig 2A). The same significant pattern was observed in heat ramping, where the critical thermal maximum (CTmax) was 2.57°C lower for field males and 1.67°C lower for field females, compared with laboratory flies of the same sex reared at 25°C (Table 1; Fig 2B). No significant difference was observed between field and laboratory flies reared at 25°C in 2 h chill coma recovery time in either of the sexes (Table 1; Fig 2C). However, the cold ramping assay showed a significantly lower critical thermal minimum (CTmin) for field flies (females: 0.62°C; males: 0.74°C), compared to laboratory flies reared at 25°C (females: 6.19°C; males: 5.92°C) (Table 1; Fig 2D). We found small but significant differences between sexes in the field flies in the heat knock down, heat ramping and 2 h chill coma recovery assays (Table 1; Fig 2). There was no differentiation between sexes in the laboratory flies reared at 25°C (Table 1; Fig 2).

**Table 1:** Thermal tolerance analysis.

Trait	sex	*Md*_*lab25—field*_	*P*-value	treatment	*Md*_*males—females*_	*P*-value
static heat knockdown	males	87.07 min	< 0.001***	field	-7.85 min	< 0.05*
	females	111.13 min	< 0.001***	laboratory	-31.9 min	0.39
heat ramping	males	2.57°C	< 0.001***	field	-0.79°C	< 0.05*
	females	1.67°C	< 0.001***	laboratory	0.11°C	0.29
2 h chill coma recovery	males	-2.12 min	0.07	field	3.15 min	< 0.05*
	females	1.70 min	0.20	laboratory	-0.65 min	0.59
cold ramping	males	5.18°C	< 0.001***	field	0.12°C	0.44
	females	5.57°C	< 0.001***	laboratory	-0.27°C	0.06

Analyses of the difference between field and laboratory flies reared at 25°C within sexes and of differences between males and females within treatments in four thermal tolerance assays. Results are presented as differences in the median (Md). Analyses were performed with randomization tests (*n* = 100,000).

* P < 0.05,

*** P < 0.001. Results from the analyses of laboratory flies reared at 13°C are presented in the text.

Laboratory flies reared at 13°C were submitted to cold ramping and heat ramping. The CTmin of females and males (medians, Md) reared at 13°C were comparable to that of field flies (*Md*_*lab13—field*_ = 0.03; *P* = 0.96 and *Md*_*lab13—field*_ = 0.25; *P* = 0.65, respectively), but were significantly different from laboratory flies reared at 25°C (*Md*_*lab13—lab25*_ = -5.54; *P* < 0.001 and *Md*_*lab13—lab25*_ = -4.93; *P* < 0.001, respectively) (Fig 2D). The same picture emerged from the heat ramping assay, in which the CTmax of females and males reared at 13°C did not differ from the CTmax of field flies (*Md*_*lab13—field*_ = 0.32; *P* = 0.53 and *Md*_*lab13—field*_ = 0.26; *P* = 0.68, respectively), but differed significantly from the CTmax of laboratory flies reared at 25°C (*Md*_*lab13—lab25*_ = -1.35; *P* < 0.01 and *Md*_*lab13—lab25*_ = -2.31; *P* < 0.001, respectively) (Fig 2B).

### Egg-to-Adult Viability

**Fig 3:**
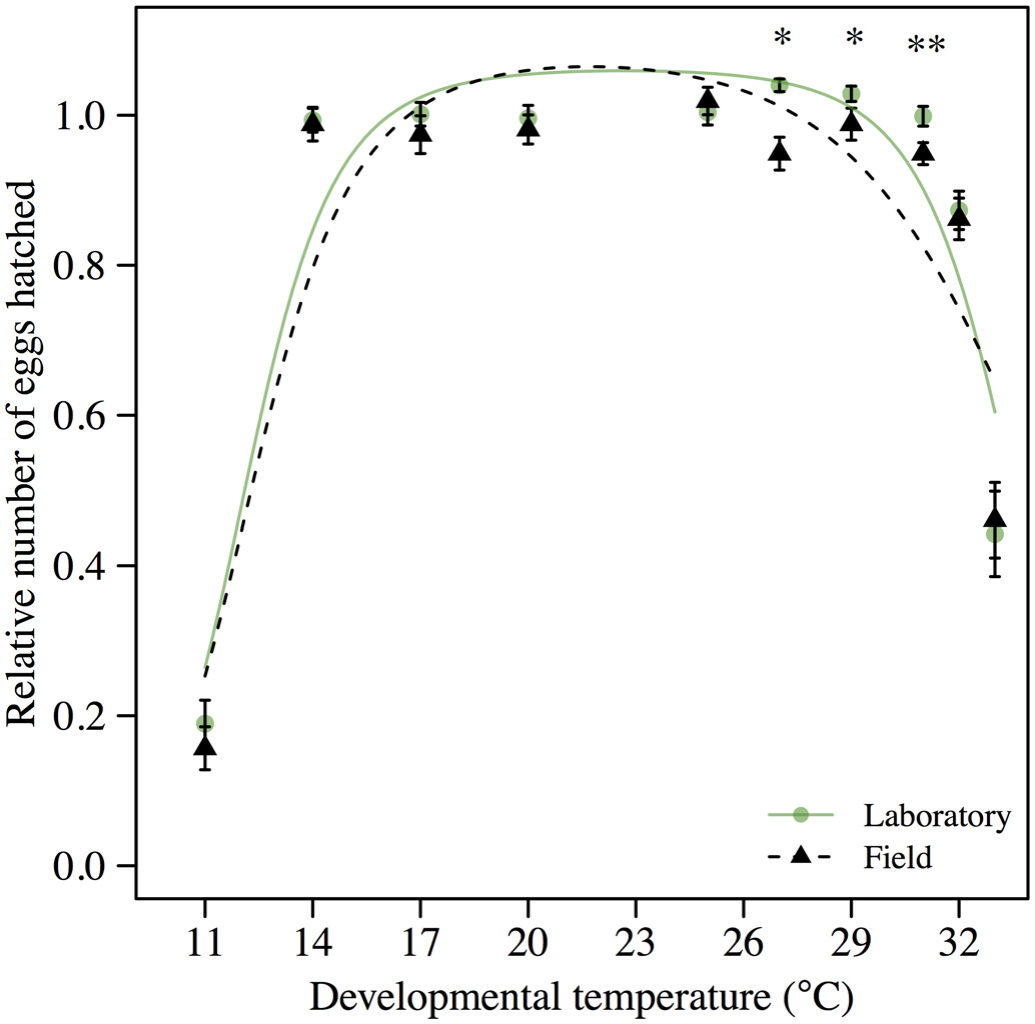
Egg-to-adult viability across developmental temperatures. Mean egg-to-adult viability ± s.e. for field and laboratory reared flies across a range of constant developmental temperatures. We assessed egg-to-adult viability with laboratory flies after three generations in the laboratory and with freshly collected field flies. The performance curves obtained from the logistic regression are represented with a solid and a punctuated line for laboratory and field flies, respectively. The performance curves, as well as the plotted values, are standardized to the average performance at 20 and 25°C within each treatment. * P < 0.05, ** P < 0.01.

We obtained thermal performance curves for egg-to-adult viability (from 11°C to 33°C) of eggs collected from laboratory flies reared at 25°C and the field flies. We found a significant interaction between developmental temperature and acclimation treatment (*F*_3,385_ = 4.53, *P* < 0.01). We detected no difference between the two acclimation treatments in egg-to-adult viability at low temperatures (11°C, 14°C and 17°C; Table 2; Fig 3). Instead the interaction was driven by a lowered egg-to-adult viability of field flies compared to laboratory flies at high temperatures (27, 29 and 31°C; Table 2; Fig 3).

**Table 2:** Egg-to-adult viability analysis.

developmental temperature (°C)	11	14	17	27	29	31	32	33
*Md_lab25—field_*	0.049	-0.017	0.037	0.037	0.037	0.066	0.017	-0.019
*P*-value	0.48	0.34	0.35	< 0.05*	<0.05*	<0.01**	0.63	0.67

Analysis of the difference in egg-to-adult viability between field and laboratory flies at different developmental temperatures using randomization tests (*n* = 100,000).

* P < 0.05,

** P < 0.01.

## Discussion

In this study we investigated the extent to which *D*. *melanogaster* acclimatize to the thermal winter conditions in the field in temperate Denmark and if this acclimatization can be replicated in the laboratory. We further aimed at assessing whether the winter acclimatization resulted in any costs in terms of decreased heat tolerance, and if the acclimatization had cross-generational fitness effects. Flies collected in the field had a CTmin that was 5.57 C lower for females and 5.18°C lower for males, compared to flies developed at 25°C in the laboratory. Thus winter acclimatization results in strong benefits in terms of increased cold tolerance of adult flies. However, winter acclimatized flies also had a reduced heat tolerance compared to laboratory flies developed at 25°C; i.e. high costs in terms of decreased heat tolerance are associated with winter acclimatization in the field. We also found cross-generational costs of acclimatization to winter conditions in terms of reduced egg-to-adult viability at high temperatures of eggs laid by field caught females relative to eggs laid by flies reared in the laboratory at 25°C (Fig 3). However, no cross-generational benefits of winter acclimatization were observed at low temperatures (Fig 3). Many other biotic and abiotic factors than temperature differ between field and laboratory reared flies: e.g. parasitic load, diet composition and availability, and humidity. Thus to provide further evidence for our claim that the costs and benefits induced by winter acclimatization can be partly ascribed to temperature, we also let flies develop in the laboratory at 13°C, and compared cold and heat tolerance of these flies with those developed at 25°C in the laboratory and the field flies. Results showed increased cold tolerance and decreased heat tolerance, compared to flies reared in the laboratory at 25°C, but similar tolerance levels to those observed in field flies (Fig 2B and 2D). This suggests that the effects of winter acclimatization observed in heat and cold tolerance of field flies, are mainly a consequence of low developmental temperatures.

Fitness benefits of thermal acclimation have been shown in numerous studies on ectotherms [4,6,8,30,31]. However, benefits and potential costs of thermal acclimation have rarely been studied on animals acclimatized in the field. In the Northern hemisphere *D*. *melanogaster* is likely to overwinter as adults in refugia (e.g. small caves, crevices and cellars) with slightly elevated temperatures [29,32]. Thus cold tolerance of adults may constrain the northern boundaries of the distribution of this species. The effect of acclimatization in *D*. *melanogaster* to thermal conditions during winter in Denmark observed in this study is rather dramatic. CTmin of winter acclimatized flies (0.62°C in females and 0.74°C in males) is lower than observations in any other study on this species that we are aware of (S1 Table), suggesting an enormous capacity of *D*. *melanogaster* to adjust its physiology to ambient temperatures and thereby survive the winter in a suitable habitat. Other species of *Drosophila* such as *D*. *montana* and *D*. *triauraria* are known to increase their cold tolerance at winter through reproductive diapause [33,34]. In contrast *D*. *melanogaster* show a somewhat shallow reproductive diapause (or quiescence) [35,36], and it has not been investigated whether this may increase cold tolerance in females.

We observed large costs of winter acclimatization in terms of decreased heat tolerance (Fig 2C and 2D). Winter acclimatized flies had a CTmax that was 1.67°C lower in females and 2.57°C lower in males than for flies reared in the laboratory at 25°C, and their time to coma onset at 37°C was 111 and 87 min shorter for females and males, respectively (Table 1). However, testing flies developed in the laboratory at low temperatures (13 C) and using ecologically relevant thermal ramping assays, revealed similar results to those observed when using winter acclimatized flies (Fig 2B and 2D). The winter acclimatization responses identified in this study, are primarily driven by changes in temperature and can thus be approximated through ecologically relevant rearing and testing schemes in the laboratory.

The two measures of cold tolerance used in this study; the ramping assay and the 2 h chill coma recovery assay, are widely used in ecophysiological studies on thermal tolerance in ectotherms and both have been suggested to be ecologically relevant measures of cold tolerance [4,37,38]. We expected the winter-acclimatized flies to have an increased cold tolerance. However, the two measures of cold tolerance gave very different results; strong benefits of winter acclimatization were observed in CTmin (Fig 2D), while no benefits were observed in 2 h chill coma recovery time (Fig 2C). Previous studies have shown low correlations between chill coma recovery time and other measures of cold tolerance, as well as between chill coma recovery time and climatic variables [39,40]. The acclimation responses of cold resistance measures are highly complex and correlates poorly when assessed across different acclimation schemes [41]. Furthermore, recovery time is not only dependent on the ability of flies to recover full muscle control, but also on the extent to which muscle resting potentials have been perturbed, which in turn relies on the duration and temperature of the cold exposure [38,42]. Thus both low ecological relevance and details in the methodological approach may explain the lack of a winter acclimatization response in 2 h chill coma recovery time in this study.

**Fig 4:**
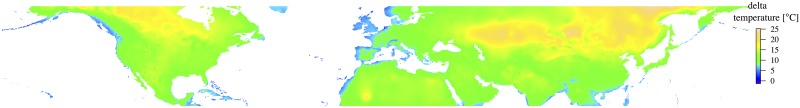
Differences between average temperatures in early spring (March) and the maximum temperatures the following month (April). The temperatures experienced during the adult stage of *D*. *melanogaster* in nature may differ from the temperatures experienced during development. To quantify these temporal changes in temperature, we calculated the differences between average temperatures in March and the maximum temperatures in April in the Northern hemisphere (delta temperature (°C)). Temperature data was obtained from WorldClim and are interpolations of data from 1950–2000 [53]. The map was produced with the raster package in R vers. 2.2–5 [54].

Cross-generational effects are an important, and often neglected, component of stress tolerance of natural populations [27]. However, when cross-generational effects are important, environmental changes between generations, e.g. unpredictable changes in temperature, may result in plastic responses becoming maladaptive [43–45]. We observed cross-generational costs of winter acclimatization in egg-to-adult viability at high temperatures in the field flies. This may have consequences for ectotherms developing and producing eggs at low temperatures, e.g. in early spring, if they subsequently are faced with a sudden increase in temperature. To quantify whether temporal changes in temperatures in the spring are in a range, which may affect the fitness outcome of acclimation, we calculated the differences between average temperatures in early spring (March) and the maximum temperatures the following month (April) in the Northern hemisphere. Large areas of the Northern hemisphere have increases in temperature between the two months in the proximity of 15°C (Fig 4). This simplistic approach does not integrate the microclimates which may serve as suitable habitats [30], nevertheless it emphasizes that large climatic changes on a short timescale occur in nature. Many studies predict future distributions of ectotherms based on CTmax, CTmin and thermal performance curves, which are estimated from ectotherms acclimated to benign laboratory conditions [9,46–51]. A recent study on *Drosophila subobscura* found large increases in both cold and heat resistance of flies sampled in Denmark during winter [52]. The trade-off between heat and cold resistance as a result of winter acclimatization in *D*. *melanogaster* observed in this study, may therefore not be a general phenomenon, but be species specific, and depend on ecological components such as overwintering strategies. Large differences in the acclimatization response between species coupled with the seasonal change in temperature will affect the precision of models predicting future distributions of ectotherms based on CTmax and CTmin.

## Conclusions

Based on our results we advocate for in depth assessments of the phenotypic plasticity of thermal tolerance breadths under a range of field conditions or ecologically relevant laboratory conditions in as many species as possible. Assuming that natural populations are acclimatized to the environment in which they are situated, distribution models should use CTmin and CTmax of cold and heat acclimated individuals, respectively. This approach may improve the accuracy of the models, without increasing the complexity. This is crucial for understanding the consequences of climate change on ectotherms, and is necessary for an understanding of how an average warmer and more variable future climate will interact with the acclimatization (phenotypic plasticity) of ectotherms in shaping their future distributions.

## Materials and Methods

### The Natural Habitat

Field *D*. *melanogaster* for immediate phenotypic assessments and for establishment of a fresh laboratory strain were collected at Karensminde orchard (permission granted by owner Bendt Rokkjær Olsen) on the Danish peninsula of Jutland (55°56'42.46"N, 10°12'45.31"E). Temperature data in the air and within and above the compost heap (Fig 1) were collected with data loggers (iButton Data Loggers, Maxim, Sunnyvale, California, USA) every half hour and extracted with the software OneWireViewer (Maxim, Sunnyvale, California, USA). The low air temperatures prevented the field flies from flight during collection, thus all field flies were collected from the surface of the heap with aspirators.

### Establishment of Laboratory Strain

In November 2013 we collected 25 inseminated *D*. *melanogaster* females from the compost heap. The females were maintained in the laboratory at 25°C in a 12:12 L:D photoperiod, where they produced eggs in individual vials containing 7 mL standard oatmeal-sugar-yeast-agar *Drosophila* medium (the medium used throughout the experiment). Five male offspring and five female offspring from each of the 25 field females (125 males and 125 females) were pooled to establish a mass bred laboratory strain. The laboratory strain was maintained at 25°C in a 12:12 L:D photoperiod throughout the experiment at a population size above 500 in each generation.

To address potential confounding factors, e.g. diet composition, which differ between field and laboratory flies, we reared another set of laboratory flies from the same population at 13°C (which corresponds roughly to the temperature at the bottom of the pile prior to testing of field flies, see Fig 1) for one generation and subsequently submitted them to heat and cold ramping starting from 20°C (described below). The flies were density-controlled during development (40 eggs per plastic vial with 7 mL medium) and three days old at the time of the testing.

### Thermal Tolerance Assays

The temperature tolerance of the flies from two treatments (laboratory flies reared at 25°C and field flies) were compared using four stress tolerance assays: 2 h chill coma recovery, cold ramping from 18°C, static heat knockdown and heat ramping from 18°C (described below). The field flies were collected at the day of the experimental assays (17^th^ of December), and maintained at approximately 15°C during one hour of transportation (except for flies used in the 2 h chill coma recovery assay—see below), and at 11°C in the laboratory prior to each assay. Flies from the laboratory treatment were three days old, density-controlled during development (40 eggs per plastic vial with 7 mL medium) and had experienced two generations in the laboratory prior to testing, whereby laboratory adaption is expected to be limited. Factors such as diet composition, parasite load and age may differ between laboratory and field flies, and cannot be properly controlled or quantified under field conditions. Twenty-five males and 25 females from the laboratory and field treatments were assessed for their tolerance in each of the four assays. The flies were sexed by eye without the use of anesthesia.

The 2 h chill coma recovery assay was set up at the collection site. Laboratory flies reared at 25°C were brought to the field in an insulated Styrofoam box at a temperature of 20±3°C. In the field, newly caught flies and flies from the laboratory were transferred individually to small thin-walled glass vials (45×15 mm), which were closed with plastic lids and submerged in ice water for two hours. The temperature in the ice water was monitored with data loggers, and measured to 0°C. While being submerged in ice water, flies were transferred to the laboratory and after a total of two hours of exposure to 0°C, flies were allowed to recover at 22°C. The time until a fly was standing upright on all legs was scored as the 2 h chill coma recovery time.

The static heat knockdown assay was initiated by submerging small glass vials (same type as described above) containing individual flies from the two treatments (laboratory flies reared at 25°C and field flies) into 37°C water [55]. The state of the flies was monitored as for CTmin and CTmax (see below); the time at which flies did no longer react when provoked with strong light or mechanical disturbance was defined as the heat knock down time.

The CTmin of the field and two laboratory treatments (13 and 25°C) was assessed using a cold ramping assay. Here flies were transferred individually to small glass vials (same type as described above), which were attached to a rack and submerged into an antifreeze / water mixture [39,56]. Hereafter the temperature of the water was decreased by 0.1°C / min. In the experiments we continuously monitored the state of individual flies by gently knocking on the vials with a metal stick and illuminating the vials with a flashlight. When a fly failed to move, e.g. the proboscis or a leg in response to strong light or due to the mechanical disturbance, the water temperature was registered and used as a proxy for the temperature of coma onset (CTmin). Heat ramping was performed using the same approach as for cold ramping, but with an increase in temperature of 0.1°C / min [20,57]. The state of the flies was monitored as for CTmin; the temperature at which flies did no longer react when provoked with strong light or mechanical disturbance was defined as the coma onset (CTmax).

### Egg-to-Adult Viability

Egg-to-adult viability of eggs from laboratory flies reared at 25°C and field flies was assessed at temperatures 11, 14, 17, 20, 25, 27, 29, 31, 32 and 33°C. We used eggs from flies which had experienced three generations of laboratory rearing and eggs from field flies kept at 25°C in a 12:12 L:D photoperiod for two days prior to producing the eggs used in the experiment. Apart from different temperatures, the parental field and laboratory flies had experienced different food composition, parasite loads etc. To ensure random sampling of eggs and to reduce transfer of field microorganisms, we used an egg collection method with standard medium and 3% agar [58]. The parental flies from each treatment produced eggs in two 300 mL bottles each containing 50 mL medium and a pile of yeast-paste. Eggs were washed from the surface of the medium, randomized and transferred to a filter paper [58]. Thereafter eggs were transferred from the filter paper to vials, such that 20 vials containing 20 eggs were created for each temperature per parental acclimation treatment. The number of emerging flies was counted, and the average fraction of eggs reaching adulthood is reported.

### Data Analysis

Assumptions of normality of residuals and homogeneity of variances were not fulfilled in data from the stress tolerance assays. Transformations did not change this. Furthermore, we observed violations of assumptions for standard non-parametric methods (e.g. large differences in the skew and variances), and we therefore used randomization tests for the analysis. We used the difference between the medians (*Md*) of the two groups being compared (i.e. *Md*_*laboratory*_ − *Md*_*field*_ = *Md*_*lab-field*_) as the test statistic. We performed 100,000 permutations for each comparison to obtain the null distribution of the test statistic. The observed difference in medians was compared to the null distribution and a two-tailed test was performed to obtain a *P*-value.

To assess if changes in egg-to-adult viability across developmental temperatures were dependent on the parental acclimation treatment of the flies, we used a logistic regression. The model included temperature as a cubic continuous variable, treatment and their interaction. We detected overdispersion in our model and corrected the standard errors using a quasi-generalized linear model [59]. Models were compared using F-tests, first for the full model described above against a null model, and in the case of a significant full model, we compared the full model with a reduced model in which the interaction term was omitted. We found a significant interaction between developmental temperature and treatment, and halted model reduction. We are, however, cautious as to make conclusions from the significant interaction, as we observed lower egg-to-adult viability of field flies compared to laboratory flies at benign temperatures (20°C and 25°C) (*F*_1,76_ = 5.92, *P* < 0.05). To ensure that the interaction between developmental temperature and treatment was driven by different shapes of the two curves and not an artefact of a general lower egg-to-adult viability of field flies compared to laboratory flies reared at 25°C in the experiment, we standardized survival by the average survival observed at the two benign temperatures 20°C and 25°C within each treatment. We could then compare relative egg-to-adult viability at high (27, 29, 31, 32, 33°C) and low (11, 14 and 17°C) temperatures. Standardized data were unsuitable for logistic regression and also violated assumptions of normality. We therefore tested for differences in egg-to-adult viability between field and laboratory flies at each temperature (benign temperatures omitted), using randomization tests with the same approach as in the analysis of stress tolerance data. All statistical analyses were performed in R vers. 3.1.0 [60].

## Supporting Information

S1 Table CTmin estimates across studies. The CTmin estimates included in this table are solely those that have been estimated using the approach used in our study. Scoring methods: (a) inability to maintain an upright posture; (b) unable to move any body part.
(DOCX)
