# Supplementary material for: Strong Costs and Benefits of Winter Acclimatization in Drosophila melanogaster
Source: PLoS One. 2015 Jun 15;10(6):e0130307. doi: 10.1371/journal.pone.0130307 (PMC4468168; doi:10.1371/journal.pone.0130307)
Supplement: S1 Table — The CTmin estimates included in this table are solely those that have been estimated using the approach used in our study. Scoring methods: (a) inability to maintain an upright posture; (b) unable to move any body part. (DOCX) [file pone.0130307.s001.docx]

**S1 Table CTmin estimates across studies**

The CTmin estimates included in this table are solely those that have been estimated using the approach used in our study. Scoring methods: (a) inability to maintain an upright posture; (b) unable to move any body part.

| CTmin (°C) | Study | ramping speed (°C/min) | start temperature (°C) | adult acclimation (°C) | developmental acclimation (°C) | sex | scoring method |
| --- | --- | --- | --- | --- | --- | --- | --- |
| 3.7 | (1) | 0.1 | 20 | 15 | 20 | Female | (a) |
| 4.0 | (1) | 0.25 | 20 | 15 | 20 | Female | (a) |
| 4.4 | (1) | 0.5 | 20 | 15 | 20 | Female | (a) |
| 4.7 | (1) | 0.1 | 20 | 20 | 20 | Female | (a) |
| 4.9 | (1) | 0.25 | 20 | 20 | 20 | Female | (a) |
| 5.3 | (1) | 0.5 | 20 | 20 | 20 | Female | (a) |
| 6.3 | (1) | 0.1 | 20 | 25 | 20 | Female | (a) |
| 6.8 | (1) | 0.25 | 20 | 25 | 20 | Female | (a) |
| 6.7 | (1) | 0.5 | 20 | 25 | 20 | Female | (a) |
| 2.4 | (2) | 0.1 | 20 | 15 | 15 | Female | (b) |
| 3.1 | (2) | 0.1 | 20 | 15 | 15 | Male | (b) |
| 3.3 | (2) | 0.1 | 20 | 20 | 20 | Female | (b) |
| 5.0 | (2) | 0.1 | 20 | 20 | 20 | Male | (b) |
| 2.8 | (3) | 0.1 | 23 | 19 | 19 | Female | (b) |
| 3.9 | (3) | 0.1 | 23 | 13-25 | 13-25 | Female | (b) |
| 6.6 | (3) | 0.1 | 23 | 27 | 27 | Female | (b) |
| 5.8 | (3) | 0.1 | 23 | 25-29 | 25-29 | Female | (b) |
| 3.5 – 4.8 | (4) | 0.1 | 24 | 24 | 24 | Female | (b) |
| 2 | (4) | 0.1 | 24 | 8-27 | 8-27 | Female | (b) |
| 4.6 | (5) | 0.1 | 25 | 24 | 24 | Female | (b) |
| 1.7 | (5) | 0.1 | 25 | 17 | 17 | Female | (b) |
| 2 | (5) | 0.1 | 25 | 12 – 22 | 12 – 22 | Female | (b) |
| 2.4 | (5) | 0.1 | 25 | 8 – 27 | 8 - 27 | Female | (b) |
| 1.5 | (6) | 0.1 | 10 | 15 | 15 | Both | (a) |
| 2.5 | (6) | 0.1 | 10 | 18 | 15 | Both | (a) |
| 3.5 | (6) | 0.1 | 10 | 21 | 15 | Both | (a) |
| 4.5 | (6) | 0.1 | 10 | 25 | 15 | Both | (a) |
| 5 | (6) | 0.1 | 10 | 19 | 15 | Both | (a) |

1. Chown SL, Jumbam KR, Sørensen JG, Terblanche JS. Phenotypic variance, plasticity and heritability estimates of critical thermal limits depend on methodological context. Funct Ecol. 2009;23:133–140.

2. Kristensen TN, Loeschcke V, Bilde T, Hoffmann AA, Sgró C, Noreikienė K, et al. No inbreeding depression for low temperature developmental acclimation across multiple *Drosophila* species. Evolution. 2011;65:3195–3201. doi:10.1111/j.1558-5646.2011.01359.x

3. Overgaard J, Kristensen TN, Mitchell KA, Hoffmann AA. Thermal tolerance in widespread and tropical Drosophila species: does phenotypic plasticity increase with latitude? Am Nat. 2011;178:S80–S96. doi:10.1086/661780

4. Overgaard J, Hoffmann AA, Kristensen TN. Assessing population and environmental effects on thermal resistance in *Drosophila melanogaster* using ecologically relevant assays. J Therm Biol. Elsevier; 2011;36:409–416. doi:10.1016/j.jtherbio.2011.07.005

5. Kristensen TN, Overgaard J, Hoffmann AA, Nielsen NC, Malmendal A. Inconsistent effects of developmental temperature acclimation on low-temperature performance and metabolism in *Drosophila melanogaster*. Evol Ecol Res. 2012;14:821–837.

6. Gibert P, Huey RB. Chill-coma temperature in *Drosophila*: effects of developmental temperature, latitude, and phylogeny. Physiol Biochem Zool. 2001;74:429–434. doi:10.1086/320429
